# Testing a strength and conditioning program to prevent common manipulative technique training injuries in chiropractic students: a study protocol for a randomised controlled trial

**DOI:** 10.1186/s12998-018-0192-0

**Published:** 2018-06-28

**Authors:** Christopher J. Hodgetts, Bruce F. Walker

**Affiliations:** School of Health Professions, Murdoch University, Perth, Western Australia

**Keywords:** Prevention, Strength and conditioning, Chiropractic, Manual therapy, Injury

## Abstract

**Background:**

Spinal manipulation is the primary therapy utilised by chiropractors in the management of their patients. The skills required may feel foreign to chiropractic students as they need strength and endurance in movement patterns they may not have otherwise been exposed to. This may lead to injury while learning manipulative techniques. It is plausible to suggest that the implementation of a strength and conditioning program early in a practitioner’s career could reduce the incidence and progression of injuries. The study aims to test the effectiveness of a strength and conditioning program in reducing the risk of chiropractic students’ acquiring injuries while learning the skill of spinal manipulation.

**Methods:**

This study will involve a prospective cohort of chiropractic students who are currently learning manual therapy at an undergraduate level. Participants will be eligible for inclusion if they are enrolled in 3rd or 4th-year chiropractic manual therapy units at Murdoch University chiropractic course. The intervention group will follow a 12-week strength and conditioning program comprised of preventative exercises that address each body region previously identified as being prone to injury. The control group will complete a 12-week walking program. The primary outcome is injury rate, measured via a short questionnaire. The secondary outcome will be strength, measured via submaximal strength tests.

**Discussion:**

The prescribed exercises are aimed at improving the strength and endurance of those muscle groups involved in commonly taught manual therapy tasks. The resistance bands have been chosen as they are inexpensive, simple to implement for the purposes of the study, and acceptably safe. A video format was selected to allow ease of access for participants, provide a detailed description and a visual representation of the exercises to be performed. A questionnaire was designed as a means to assess the influence of the strength and conditioning program on injury rate and the impact this may have on the students’ ability to continue practicing. The International Physical Activity Questionnaire has been chosen to measure the participants level of activity before beginning the exercise program.

**Conclusion:**

This research protocol will be the first large-scale study to investigate the effectiveness of a strength and conditioning program to reduce injuries within chiropractic students learning manual therapy.

**Trial registration:**

Australia New Zealand Clinical Trials Registry (ACTRN12617001638325p).

**Electronic supplementary material:**

The online version of this article (10.1186/s12998-018-0192-0) contains supplementary material, which is available to authorized users.

## Background

Spinal manipulation is the primary therapy utilised by chiropractors in the management of their patients [1]. The skill required to deliver an effective manipulation takes several years of training at a tertiary institute. The motor control and movement patterns required may feel foreign to students, leading to imperfect technique and sometimes injury [2]. Among student and early career chiropractors, injury rate has been shown to range from 31 to 56% [1–5]. A retrospective survey of students from the Canadian Memorial Chiropractic College (CMCC) revealed that reports of adverse reactions were highest in the second year of the course when many techniques were initially taught [4].

The most injured body regions seem to vary within the literature, but common areas include the shoulders, wrists, neck and the lower back [3]. Of these, the most common forms of injuries were ligament sprain, tendonitis and muscular strain. It is important to note that some studies did not separate those injuries that occurred while delivering a technique and those that happened while receiving it. However, because chiropractors appear at risk of injury within their first five years of practice, rather than only during their education, delivering a manipulation is likely to be at least part of the reason for becoming injured [3].

Literature suggests that different population subsets had a greater risk of developing complications in specific areas. Females were found to have an increased risk of lower back, wrist and shoulder injuries, while males were more likely to develop injuries to the neck [2]. Almeida et al. [6] suggested that females report training injuries more frequently than males, but they comment that this could be attributable to disparities in symptom reporting rather than a true difference in injury rate.

There is currently conflicting evidence as to whether a higher body mass index (BMI) increases the risk of developing an injury during manual therapy training within a chiropractic program. Ndetan et al. [5] established that students who were overweight or obese were more likely to report an injury affecting the lower back while receiving a manipulation and a neck or shoulder injury while delivering one. However, Holm and Rose [3] suggest there is no association between, age, height, or weight and developing an injury while learning manual therapy skills. They proposed that it is physical capabilities rather than body measurements that contribute to training-related injuries in student chiropractors.

The high injury rate in the first five years of practice suggests that new chiropractors may not have the necessary strength and conditioning or use less than optimal biomechanics when manipulating [3]. It appears likely that those chiropractors in the early stages of their career begin to modify their technique to reduce mechanical stress on their bodies. Holm and Rose [3] suggest that optimal biomechanical positions should be promoted with students on an individual basis.

Manipulative techniques taught within chiropractic colleges often require awkward postures such as bending, stooping and rotating while simultaneously applying forces [2]. Chiropractors are subjected to these dynamic motions on a regular basis, which may result in undesirable biomechanical loading and subsequent injury [7]. This may be additionally true for chiropractic students due to their inexperience and increased time under load while they refine their technique including positioning, anatomical contacts, and delivery of the manipulative force.

In addition to this, Stock [8] identified a causal relationship between musculoskeletal conditions of the hand and wrist with repetitive forceful work. A more recent study suggested that force is the primary ergonomic risk factor for hand disorders amongst manual workers, though repetition may play a role. These findings may have implications for those who undertake repetitive forceful manipulations. Interestingly, there is little evidence to suggest that working with the hand in a non-neutral position is a risk factor for wrist and hand injury [9].

Injuries to chiropractic students and chiropractors early in their professional careers could have a significant effect on their longevity in the profession and affect their personal life. Depending on the severity and chronicity of an injury, ramifications could include; adverse impact on skills mastery, earning potential, quality of life, and even impact psychological well-being. Therefore, it is in the best interest of both students, educational and accrediting bodies to reduce the risk of personal injury to chiropractic students and chiropractors.

Our literature search failed to reveal any strength and conditioning program for chiropractic students to prevent injury while learning manipulative techniques or in the first years of practice. The concept of a strength and conditioning plan with the aim of reducing the likelihood of injury is worth considering as the implementation of such a program early in a practitioner’s training and career could reduce the incidence and progression of injuries. Similarly, Bork et al. [10] suggested that “specific strategies should be developed to reduce work-related musculoskeletal disorders in the practice of physical therapists”. In the 15 years since that study was published, researchers are yet to explore avenues to reduce the prevalence of injuries amongst manual therapists in training. The purpose of this research protocol is to develop a plan for a large-scale study to examine the effectiveness of a strength and conditioning program to prevent injuries related to manual therapy training and practice.

## Methods

### Study design

A randomised controlled trial of chiropractic students at Murdoch University using a preventative strength and conditioning program to prevent injury. This randomised controlled trial protocol complies with the recommendations of the SPIRIT guidelines [11].

### Participants

#### Study setting

This study will take place at Murdoch University, within the School of Health Professions.

#### Recruitment

Participants will be recruited from the 3rd and 4th year Chiropractic Science cohort at Murdoch University when manual therapy training is at its most intense. Students will be informed of the study and recruited through word of mouth, direct email and student Facebook groups.

#### Inclusion criteria

Potential participants will be either female or male students 18 years or older studying chiropractic on either a full or part-time basis.

#### Exclusion criteria

Potential participants will be excluded from this study if they are currently suffering from musculoskeletal pain in the shoulder, neck or low back region or have done so in the past 3 months or have a reported chronic physical disability.

#### Sample size

Based on an approximate and conservative baseline injury rate of 30% (0.3) derived from a comprehensive review [1, 5], a sample size of *n* = 212 (*n* = 106 per group) will have 80% power to detect a decrease in injury rate of 50% to an injury rate of 15% (0.15) in the treatment group (alpha = 0.05, beta = 0.2).

#### Allocation

Random allocation will be achieved using Research Randomizer [12]. The participants will be randomised into two, equal sized block groups. The sequence will be concealed in opaque envelopes until the interventions are assigned. A member of the research team will be responsible for enrolling participants and implementing the randomisation process. The participants will not be blinded. The assessors and data analysts will be blinded to which group the participants are allocated to.

### Intervention

Intervention group participants will undergo an individual, unsupervised 12-week strength and conditioning program which aims to improve strength and endurance of the musculature around the joints identified above. Exercise instructions are detailed below and will use mostly elastic resistance bands [13]. The program will require three repetitive sets for each exercise as this has been demonstrated to produce the most effective strength gains [14]. The program will be undertaken three days per week, which lies in an optimal training range [14]. The sets and repetitions for each exercise are based on novice and intermediate endurance training [15]. The length of the program is based on results that have shown a significant change in muscle strength and size after a 12-week exercise regime [16] (Table 1). It should be noted that this particular study focussed on a free-weight training program, but 12-week programs have been used with TheraBand resistance previously and are documented in the literature [17, 18]. The intensity of the exercises can be progressed by shortening the band or changing to a higher resistance band, and thus increasing the demands of the activity. Where exercises do not use elastic bands, the load can be progressed through increasing the duration of the exercise.

**Table 1:** Exercise program parameters

Parameter	
Tempo	3-s eccentric, 1-s hold, 3-s concentric (3–1-3)
Repetitions	15
Intensity	13/20 Rate of Perceived Exertion (RPE) [37]
Sets	3
Rest	1 min
Frequency	3 days per week
Duration	12 weeks

#### Feasibility study

We conducted an initial feasibility study around the design of region-specific exercises that hypothetically strengthen and improve the endurance of the at-risk anatomical areas of the shoulder, wrist and low back described above. Video clips and written instructions for each exercise of the protocol were developed and are described below. These will be supplied to the participants.

#### Instructions for participants

These exercises will be in addition to any regular exercise regime. An exercise diary will be completed by those in the active and controls groups. Furthermore, they will be required to report the dates and duration of their specific exercises below in an exercise diary provided, in order to monitor and keep record of compliance.

### **A. SHOULDER EXERCISES:** (Additional file 1) [19–21]

Due to the complexity of the shoulder joint and surrounding musculature, different exercise sessions will be prescribed for the shoulder training. This method aims to activate different muscles during each session and target shoulder strength as a whole.

### Session 1

#### Exercise 1. Internal rotation/external rotation

External rotation: Standing upright, attach the resistance band to a secure object at waist level. Grasp the resistance band tightly in one hand, elbow bent to 90 degrees, and shoulder turned inwards. Rotate the lower arm slowly outward against resistance as far as you can go without pain, hold for one second. Slowly return to start position and repeat. For internal rotation, being in a shoulder turn out position and perform this action in the opposing direction.

#### Exercise 2. Scapulothoracic wall slide

Tie the resistance band together to make a small loop around the wrist/forearm, stand with feet about 5 cm from the wall and forearms parallel on the wall. Keeping elbows at shoulder width apart creating tension by flaring wrists. Slowly slide your forearms up the wall as far as possible, then slowly back to start position.

### Session 2

#### Exercise 1. Push-ups

With different hand location (elbows close to body, elbows at 45 and elbows at 90) hand positioned at shoulder height. **Note:** If you cannot perform push-ups on your toes, then begin by having your knees on the ground.

#### Exercise 2. Latissimus pulldowns

In a seated position, the arms are held overhead at full extension, grasping the resistance band connected to a fixed object, the movement is initiated by pulling both elbows down and back, and completed by slowly returning to the initial position.

### Session 3

#### Exercise 1. Shoulder press

Stand with the centre of the band under your feet and one end in each hand. Bend the elbows and position the hands just above shoulder height. Push your hands above your head, pause and slowly return to the starting position.

#### Exercise 2. Seated rows

Sit on the floor with your legs slightly bent out in front. Loop the band around the soles of your feet. Start with your arms extended. Pull back with elbows entirely, pause and slowly return to the starting position.

### **B. WRIST EXERCISES:** (Additional file 2) [22, 23]

#### Exercise 1. Wrist flexion

Both exercises 1 and 2 can be performed with filled 600 mL bottles of water or the resistance band.

The back of the forearm is placed flat on a desk while you are seated. Your wrist hangs over the edge of the table as demonstrated in the video. Grasp either resistance band or bottle in a fully extended wrist position, palms facing upwards. Bring your hand up away from the ground into a flexed position, pause and then return to the starting position.

#### Exercise 2. Wrist extension

In the opposite position to the previous exercise, place the front of your forearm on table with wrist in the fully flexed position grasping resistance over the edge of the table. Against resistance and gravity, you are to raise the wrist, pause and then lower to starting position.

#### Exercise 3. Radial deviation

Stand or sit with the resistance band held between your hands. Palms facing up and wrist beginning with knuckles facing inwards. With tension on the band, move your hands away from the middle, pause and then return to the start position.

#### Exercise 4. Ulnar deviation

Stand or sit with the resistance band between hands, palms facing down and knuckles facing inwards. With tension on the band move your hands away from the middle, pause and return to the start position.

#### Exercise 5. Weighted wrist rotations

Use a dumbbell or hammer type object, with weight at one end and grip at the other. Stand or sit with shoulders relaxed, elbows flexed to 90 degrees and the dumbbell or hammer held in front of your body. With the wrist starting so your palm is facing up, rotate your forearm so that your palm then faces down (this will mean turning towards the middle), pause, and then return to the starting position. This can then be performed on the other hand.

### **C. LOW BACK EXERCISES:** (Additional file 3) [24–28]

#### Exercise 1. Crab walk

Stand with feet shoulder width apart with knees bent between 30 and 45 degrees. Loop the band around your ankles. Take a wide step sideways, applying tension to the band. Bring your trailing leg so you are in the original position and repeat for 15 steps. Repeat in the process in the opposite direction.

#### Exercise 2. Lunge

Stand with one foot on the middle of the band. Keeping your elbows bent, grip the ends of the band and hold at chest level. Place your opposite leg behind you, with your knee slightly bent. Keeping your trunk straight, bend your front knee lowering your body downwards, pause, slowly return to the original position and repeat.

#### Exercise 3. Deadlift

Stand in the middle of band with both feet about hip-width apart. Squat down, grasp ends of band and take up slack. Keep elbows and back straight and extend hips to slowly return from the squat to an upright position, pause and return to starting position.

#### Exercise 4. Back extension

Lay down on your front with your hands underneath your shoulders and elbows tucked in neatly at your side. Using your arms for a small of assistance extend your lower back by lifting your head and chest off the ground, pause and then slowly lower yourself back to the ground.

### Control group

The group participating in the strength and conditioning protocol will be compared to a control group. These participants will be required to walk briskly 3 times a week for 30 min for the duration of the program (12 weeks). This will be in addition to any regular exercise regime. Furthermore, they will also be required to report the dates and duration of their walking on the exercise diary provided, in order to monitor and keep record of compliance.

### Outcomes

#### Primary outcome

##### Injury rate

Measured via a brief questionnaire designed especially for this study, which asks about shoulder, wrist or low back pain. It will ask for information about the pain frequency, duration, intensity, if the pain began while practising their manual therapy skills, and if they were in the role of ‘doctor’ or ‘patient’ at the time. Self-reported region specific numerical rating scales are included to measure pain intensity [29]. The questionnaire will ask if this has impacted their ability to continue to practice their skills or their Activities of Daily Living (ADLs). It will be administered at the beginning and every 4 weeks during the trial, i.e. 3 times over 12 weeks. The questionnaire will be completed online at weeks 4 and 8, with SMS reminders sent to participants in these weeks.

#### Secondary outcome

##### Muscle Strength and endurance

One-repetition maximum (1RM) will be estimated via submaximal strength testing procedures. Grip strength will also be measured via a validated and calibrated dynamometer [30, 31]. These measurements will be performed at the beginning of the trial and repeated at the end.

### Data collection

#### Baseline measures

A questionnaire will be given to each participant to obtain demographic information, establish the amount they practise their manual therapy skills, and note any previous related injuries. A self-administered short form, the International Physical Activity Questionnaire (IPAQ), will also be completed in order to gain information on participant’s physical activity over the previous 7-day period. The IPAQ and manual therapy practise questionnaire will then be completed every 4 weeks to assess any change in injury status, including contemporary injury in the preceding 4 weeks and the impact that injury may be having on the participant.

Baseline maximal strength of muscle groups will be estimated using the multiple repetition test procedure (5–10 submaximal strength test) instead of a traditional 1RM test. In this test the maximum amount of repetitions measured for a particular load will be used to estimate the 1RM from the formula 1RM = load (kg)/(1.0278–0.0278 x reps) [32]. A 1RM will be calculated for strict shoulder press, bench press, seated row, Latissimus Dorsi pull down, deadlift, and back racked lunge. Participants will be instructed on correct use of equipment prior to the testing session and given time to familiarise themselves with the equipment. A general warm-up will be completed consisting of 2 min of low-intensity indoor rowing and of low-intensity indoor cycling. This will be followed by specific warm-up of 10 min of dynamic stretches incorporating movements that work through the range of motion required for the lifts to be tested [33]. The participants will be instructed to perform a warm-up set of 5–10 repetitions with a light load and given a 1 min rest period. Thereafter, 3–5 separate single attempts will be performed. Subjects will lift a weight initially 40–60% of the perceived 1RM. The increments of weight are dependent upon the effort required for the lift. The weight added will become smaller as the effort to lift the weight increases. If subjects can perform more lifts than designated by the testing protocol, they will be allowed a minimum of four minutes’ rest and then reassessed. Participants will be instructed to perform each exercise to acute muscular exhaustion or form fatigue. Any repetitions not performed with a full range of motion will not be used. When the subject can only lift the weight five to ten times, that weight and the number of repetitions will be recorded and used to estimate the 1RM.

**Fig. 1:**
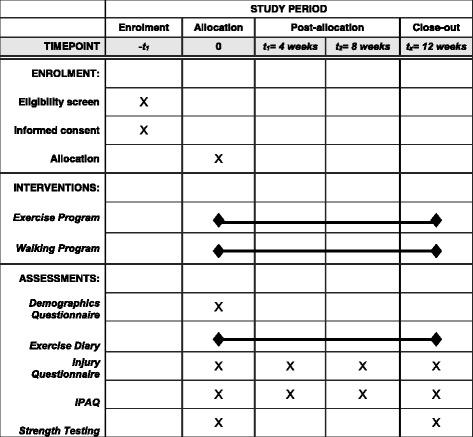
Schedule of enrolment, interventions, and assessments

Grip strength dynamometry will be performed during the strength testing procedure. Participants will be given two attempts to familiarise themselves with the device. Then they will perform three attempts on each side with a 30-s rest between each. The highest measure will be taken for each hand [30, 31]. Fig. 1 provides a schedule of enrolment, interventions, and assessments.

#### Data management

Data will be stored on a password-protected laptop and completed paperwork kept in a locked filing cabinet for 5 years. Paperwork will be shredded and data sets will be deleted after this time.

#### Statistical analysis plan

Descriptive statistics for demographic and outcome data will be based on frequency distributions for categorical data and means and standard deviations or medians, interquartile ranges, and ranges for continuous data, as appropriate. Univariate analysis for treatment group comparisons will include χ^2^ and Fisher exact tests for categorical comparisons, and t-tests or non-parametric Mann-Whitney *U or Wilcoxon signed rank* tests for continuous outcomes. After adjusting for baseline measures, changes in injury rates and muscle strength and endurance over the course of the study will be compared between groups using repeated measures ANOVA or linear mixed models, depending on the final dataset. Data will be analysed using IBM SPSS version 24.0 (Armonk, NY) and *p*-values < 0.05 will be considered statistically significant. The IPAQ results will be used as a covariate in the analysis.

### Ethics, clinical trials registration and dissemination

Ethics approval was granted by the Murdoch University Human Research Ethics Committee on the 26th of June 2017 for the initial feasibility study (Project No. 2017/146). Further Ethics approval will be sought for this study.

Participants will be provided with an information letter and informed consent form. The letter and form will be provided and collected by a member of the research team. Participant information will be securely stored.

This clinical trial is registered with the ANZCTR (ACTRN12617001638325p).

Participants and members of the research team will be the only individuals with access to the final trial data set. Feedback will be available to participants via the school research website, and project results will be published in a peer-reviewed journal. There will be no public access to participant-level datasets.

## Discussion

The prescribed exercises are aimed at improving the strength and endurance of those muscle groups involved in commonly taught manual therapy tasks. Shoulder exercises focus on improving rotator cuff endurance, dynamic scapular control and pulling/pushing endurance. Lower back exercises aim to improve trunk muscle endurance, lumbo-pelvic motor control and lower limb endurance. Wrist exercises are aimed at providing endurance and stability of the wrist through various ranges of motion. If the muscles can perform exercise tasks for a longer period of time with sustained endurance, it is hypothesised that this will reduce the chance of injury to the body.

The resistance bands were chosen as they are inexpensive, simple to implement for the purposes of the study, and acceptably safe. Measurements will be made using the TheraBand Instruction Manual and marked on the band [34].

A video format for exercises was chosen to allow ease of access for participants, a detailed description and a visual representation of the exercises to be performed. This mode of media communication is aimed at enabling participants to mirror activities with correct detail. In combination, the access to media such as YouTube [35] may also be used and is compatible with the latest generation of smartphones and other applications which allow the fastest access to information. Accessibility of this mode is free and convenient for a student and new graduate audience and can be consulted immediately. The videos, in conjunction with audio that accurately describe each exercise and how to execute them, are aimed at reducing the likelihood of injury while performing the exercises.

The 5–10 submaximal strength test was considered a safer way to assess strength and is proposed to provide an accurate estimate of 1-RM in young and active men and women [36]. The participant questionnaire was designed as a means to assess the influence of the strength and conditioning program on injury rate and the impact this may have on the students’ ability to continue practicing. The IPAQ was chosen as a way to measure the participants level of activity prior to beginning the exercise program. This allows commentary on how previous levels of exercise could impact on the outcome of completing the exercise program.

Our literature search has established that manual therapists, including chiropractic students, are at risk of musculoskeletal injury due to performance of technique and labour-intensive tasks. Research in the area of injury prevention could help to improve the overall safety and wellbeing of chiropractors, including students, and other manual therapists worldwide. If successful, a strength and conditioning program could be integrated into various curricula in an attempt to reduce injury risk amongst those students learning manual therapy skills. It could also be disseminated to the profession for their use. Such a program could supplement training on optimal biomechanics when performing manipulation and manual therapy techniques.

Future research could consider pre-class training of psychomotor skills similar to those seen in martial arts practices. These may closely reflect the rapid application of force seen in manipulative therapy, but would likely require face-to-face training rather than a home exercise program.

## Additional files

Additional file 1: Shoulder exercises. (MP4 97726 kb)

Additional file 2: Wrist exercises. (MP4 54989 kb)

Additional file 3: Lower back exercises. (MP4 71638 kb)
